# The effect of dextrose prolotherapy on patients diagnosed with knee osteoarthritis: A comprehensive systematic review and meta‐analysis of interventional studies

**DOI:** 10.1002/hsr2.2145

**Published:** 2024-06-24

**Authors:** Sorour Khateri, Fatemeh Behbahani Nejad, Farnoush Kazemi, Behnaz Alaei, Mobin Azami, Asra Moradkhani, Lobat Majidi, Yousef Moradi

**Affiliations:** ^1^ Department of Physical Medicine and Rehabilitation, Faculty of Medicine Hamadan University of Medical Sciences Hamedan Iran; ^2^ Student Research Committee Kurdistan University of Medical Sciences Sanandaj Iran; ^3^ Social Determinants of Health Research Center, Research Institute for Health Development Kurdistan University of Medical Sciences Sanandaj Iran

**Keywords:** Dextrose Prolotherapy, Evidence Synthesis, Knee, Osteoarthritis

## Abstract

**Background and Aims:**

The primary objective of this systematic review and meta‐analysis was to assess the impact of dextrose prolotherapy on individuals diagnosed with knee osteoarthritis (KOA).

**Methods:**

To conduct a thorough investigation, a variety of leading international databases were checked, including PubMed (Medline), Scopus, Web of Sciences, EMBASE (Elsevier), ClinicalTrials.gov, and the Cochrane Library. The search covered a period from January 2000 to the end of June 2023, which facilitated the collection of relevant studies.

**Results:**

The findings of the study revealed that when the studies utilizing the Western Ontario McMaster Universities Index tool (WOMAC) were combined, patients with KOA who received prolotherapy experienced an improvement in function compared with those who received other treatments (SMD: 0.20; 95% Confidence Interval [1]: −0.11, 0.51; *p* value SMD = 0.221; *I* ^2^: 78.49%; *p*_heterogeneity_ < 0.001). Additionally, there was a decrease in mean pain and stiffness among patients who received prolotherapy compared with those who received other treatments or a placebo [(SMD: −0.95; 95% CI: ‐1.14, −0.76; *p* value SMD < 0.001; *I* ^2^: 59.35%; *p*_heterogeneity_ = 0.070) and (SMD: −0.21; 95% CI: −0.32, −0.10; *p* value SMD < 0.001; *I* ^2^: 88.11%; *p*_heterogeneity_ < 0.001)]. Furthermore, based on the Visual Analog Scale (VAS) score, there was a reduction of 0.81 units out of 10 in mean pain for patients with KOA who received prolotherapy (SMD: −0.81; 95% CI: −5.63, 4.10; *p* value SMD = 0.693; *I* ^2^: 48.54%; *p*_heterogeneity_ = 0.08).

**Conclusion:**

Drawing from the data analysis performed in this meta‐analysis, it is apparent that dextrose prolotherapy exhibits promising effectiveness in reducing joint pain and stiffness, as well as improving functional performance in individuals suffering from KOA. Furthermore, it is recommended that forthcoming studies incorporate follow‐up periods to guide decisions concerning the duration of prolotherapy's effects.

## 1 INTRODUCTION

Osteoarthritis (OA) is the most common form of arthritis, posing a serious public health concern worldwide, with an increase in disability‐adjusted life years (DALY) in most countries. This cost is mostly caused by demographic shifts toward older populations and the rising prevalence of obesity. In 2017, globally, the age‐standardized point prevalence and annual incidence rate of OA were recorded at 3754.2 and 181.2 per 100,000 individuals, respectively.^1^, ^2^ OA is a multifactorial disease characterized by the progressive degeneration of joint components, particularly articular cartilage.^3^ The pathophysiology of OA is complex, involving inflammatory processes, epigenetic control, cell death pathways, and the synovial lymphatic system. Danger‐associated molecular patterns (DAMPs) and immune system activation both trigger inflammatory responses within the joint, resulting in tissue damage and inflammation. Epigenetic changes, such as DNA methylation and microRNA regulation, influence gene expression patterns involved in cartilage degradation. Joint tissue degradation is caused by a variety of cell death mechanisms, including pyro‐ptosis and autophagy. Understanding these complex molecular pathways is critical to the development of effective targeted therapies for OA.^4^ Knee osteoarthritis (KOA) is the most common manifestation, having a significant impact on both affected individuals and healthcare systems.^5^, ^6^, ^7^ KOA refers to structural changes in the knee joint that affect the articular cartilage, the subchondral bone, and other joint structures. Age, weight, heredity, and gender are all risk factors for developing KOA. KOA etiology includes a reduction in cartilage repair mechanisms with age, increased mechanical stress on joints due to obesity, a hereditary susceptibility to joint degeneration, and gender differences in joint biomechanics. Researchers hope to develop personalized treatment strategies for KOA by gaining a thorough understanding of these factors and their interactions.^8^, ^9^, ^10^, ^11^, ^12^, ^13^

The field of medical sciences has undergone significant expansion, ushering in novel treatment modalities aimed at enhancing patient outcomes and streamlining care.^14^, ^15^, ^16^ The use of injection techniques (one of the treatment modalities) in the treatment of KOA, including prolotherapy, is a topic of significant interest and ongoing research. Among the various substances used in injections for KOA, such as steroid injections, hyaluronic acid (HA), platelet‐rich plasma (PRP) injections, and prolotherapy, prolotherapy stands out as a promising alternative. Prolotherapy involves the injection of an irritant solution, typically dextrose, to stimulate the body's natural healing processes and promote tissue repair in the knee joint. While other substances like HA and PRP are also being studied for their efficacy in KOA treatment, prolotherapy has shown significant improvement in selected patients with KOA.^17^, ^18^, ^19^ The comprehensive review of literature on prolotherapy highlights its potential benefits in managing OA symptoms, particularly in the knee joint. Despite the lack of homogeneity in studies, recent trials have demonstrated that dextrose prolotherapy can effectively reduce pain scores and improve stiffness, function, and quality of life in patients with KOA.^20^, ^21^, ^22^, ^23^, ^24^, ^25^ Dextrose, a cost‐effective and readily available substance, has gained prominence as an injection‐based therapeutic option for managing chronic painful musculoskeletal conditions. Prolotherapy, which has been in use for decades, entails the injection of dextrose or other proliferants into affected tissues. The surge in interest in prolotherapy during the 1990s among both physicians and patients can be attributed to its proposed mechanism of action, centered around tissue repair and regeneration, ultimately leading to pain alleviation and enhanced functional outcomes. Given its affordability and potential to augment patient outcomes, dextrose prolotherapy has emerged as a valuable adjunct in the armamentarium of clinicians treating musculoskeletal conditions.^26^, ^27^, ^28^, ^29^ Despite extensive clinical trial investigations worldwide, the impact of prolotherapy on KOA remains contentious, with studies yielding conflicting results.^23^, ^30^ Consequently, treatment guidelines for KOA seldom endorse the use of prolotherapy, and when mentioned, it is often subject to specific conditions. Notably, guidelines from the American College of Rheumatology/Arthritis Foundation and the Osteoarthritis Research Society International (OARSI) cautiously recommend the use of dextrose prolotherapy for KOA treatment. The objective of this study is to evaluate the effect of dextrose prolotherapy on KOA patients through a systematic review and meta‐analysis approach.

## 2 METHODS

The current study followed a systematic review and meta‐analysis approach, which involved six key steps: search syntax and search strategy, screening, selection, data extraction, risk of bias assessment, and meta‐analysis. The study adhered to the guidelines outlined in the Preferred Reporting Items for Systematic Reviews and Meta‐Analyses (PRISMA) to ensure the systematic review and meta‐analysis were conducted and reported in a transparent and consistent manner.^31^

### 2.1 Search strategy and screening

The search for relevant studies in this research was conducted in several international databases, including PubMed (Medline), Scopus, Web of Sciences, EMBASE (Elsevier), ClinicalTrials.gov, and Cochrane Library. The search period spanned from January 2000 to the end of July 2023. The main keywords used in the search included “Prolotherapy,” “Dextrose Prolotherapy,” “Knee Osteoarthritis,” and “Knee Arthrosis.” Thesauruses, Emtree, and Mesh were consulted to find synonyms related to these keywords. The search syntaxes for each database were formulated using “AND” and “OR” operators based on the chosen synonyms. Additionally, a manual search was conducted by examining the reference lists of relevant articles to ensure comprehensive coverage. After retrieving the search results, the articles were imported into Endnote software version 9. Duplicate articles were identified and excluded based on matching titles, authors, and publication years. The remaining articles were then screened based on their title, abstract, and full text. The inclusion criteria for this meta‐analysis were carefully applied, and only studies that adhered to the PICOT structure (as specified in Table 1) were included. Studies that did not provide the required information outlined in Table 1 were excluded from the analysis. All aspects of the search strategy and article screening were independently performed by two authors (SKH and LM/YM).

**Table 1:** The criteria for inclusion of studies in the present meta‐analysis.

Population (P)	Intervention (I)	Comparison (C)	Outcomes (O)	Type of study (T)
People with knee osteoarthritis (KOA) were the target population for this meta‐analysis.	The desired intervention in the meta‐analysis discussed in the sources was dextrose prolotherapy with varying concentrations administered to patients with knee osteoarthritis.	The comparator group included other treatments or placebo.	Pain or its mean based on the WOMAC, VAS, and KOSS tools was the intended outcome.	Various types of clinical trial studies, including randomized controlled trials (such as parallel and crossover designs), nonrandomized controlled trials, and before‐after studies, are conducted to evaluate the efficacy and safety of desired intervention.

### 2.2 Data extraction

During this stage of the study, the following information was extracted for the meta‐analysis: authors' names, publication year, country of origin, sample size, mean age of participants, body mass index (BMI), disease duration, duration of the follow‐up period after the intervention, tools used to measure pain, characteristics of the studied population, method and type of intervention, and mean pain levels before and after the intervention. The data extraction process was carried out independently by two authors (S. K. H. and L. M.). In case of any disagreements, a third person was involved to resolve them and ensure consensus was reached on the extracted data (Y. M.).

### 2.3 Quality assessment

The quality of the study design, sampling strategy, and measurement was evaluated using the revised Cochrane risk‐of‐bias tool for randomized trials (RoB 2). For studies employing a cross‐over design, special considerations were taken into account based on the Cochrane Handbook of Systematic Reviews.^32^ Two authors independently assessed the risk of bias within each trial using Revman 5.3 software (S. K. H. and M. A./A. M.). In the event of any disagreements, consensus was reached through discussion between the two authors. If a persistent disagreement arose, a third party (Y. M.) was consulted to resolve the disagreement.

### 2.4 Statistical analysis

In this study, the desired effect size was assessed using the standardized mean difference (SMD), calculated using Cohen's method. Mean pain levels in the intervention and placebo groups before and after the intervention were extracted from the primary studies. To evaluate heterogeneity among the studies, the *I* ^2^ and *Q* Cochrane tests were employed. The Cochrane criteria were used to interpret the results, where 0%–25% indicated no heterogeneity, 25%–50% represented low heterogeneity, 50%–75% indicated high but acceptable heterogeneity, and 75%–100% indicated high and unacceptable heterogeneity. To assess publication bias, Egger's test was applied. Additionally, a meta‐regression analysis was conducted to examine the linear relationship between variables such as disease duration, duration of follow‐up after the intervention (measured in weeks), age, BMI, and the effect of prolotherapy. Meta‐regression analysis was chosen over subgroup analysis due to the limited number of studies available for subgroup analysis, which would have reduced the study's power to detect the desired relationship. Statistical analysis was performed using STATA 17.0 software, and a *p* value of less than 0.05 was considered statistically significant.

## 3 RESULTS

**Figure 1:**
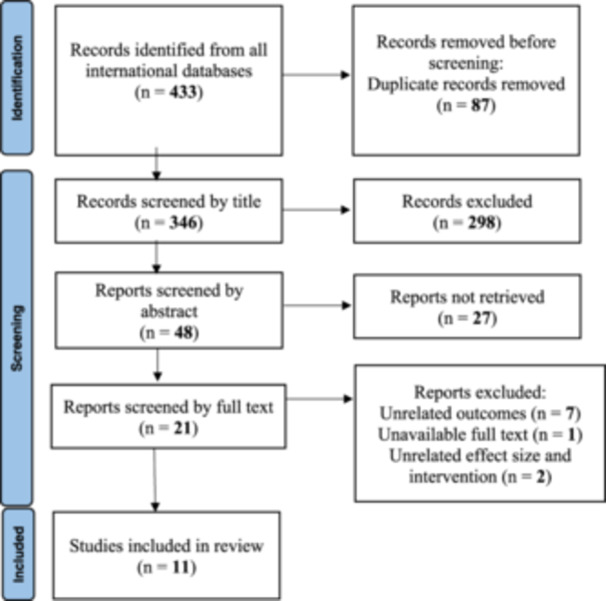
Flow diagram for related article numbers which included in meta‐analysis.

In this meta‐analysis, the initial search strategy in the selected databases yielded a total of 433 studies. After removing duplicates and screening the remaining studies based on their titles, 48 studies were deemed potentially relevant. Subsequently, after screening the abstracts, 27 articles were excluded, leaving 21 studies for full‐text screening. During the full‐text screening, seven studies were excluded from the meta‐analysis due to disparate outcomes, two studies due to differences in intervention and desired effect size, and one study due to the unavailability of the full text. Ultimately, 11 clinical trials remained for further review and analysis in the present meta‐analysis^33^, ^34^, ^35^, ^36^, ^37^, ^38^, ^39^, ^40^, ^41^, ^42^ (Figure 1).

In the clinical trials chosen, prolotherapy was the intervention employed, with varying doses of dextrose used across the trials. More precisely, four trials considered prolotherapy using 5 mL of dextrose, while one trial used 10 mL, another used 3.5 mL, one more used 2 mL, one used 6 mL, and two used 7 mL. A study did not provide specific information on the amount of dextrose used in prolotherapy. Out of the clinical trials selected, five studies included a control group using HA, two studies used a control group with PRP, three studies had a control group with normal saline, and one study included a control group with autologous conditioned serum (ACS). The Western Ontario McMaster Universities Index tool (WOMAC), Visual Analog Scale (VAS), and knee injury and osteoarthritis outcome score (KOOS) indexes were mainly utilized in the 11 chosen studies to evaluate pain and other results. Regarding postintervention follow‐up, two investigations assessed results at 24 weeks, two investigations at 52 weeks, two investigations at 12 weeks, one investigation at 18 weeks, and three investigations had a follow‐up of 10 weeks or fewer. Table 2 offers more comprehensive information.

**Table 2:** The characteristics of included randomized control trials.

Authors (Years)	Countries	Type of studies (Sample size) [follow up]	Mean age, Mean BMI, OA duration	Tools	Groups (Sample Size) [dose]	Effect Size		
						Baseline		After
Waluyo, Y. et al (2021)^33^	Indonesia	RCT (76)[12 W]	62.430.222.6	WOMAC	Prolotherapy (44)[5 mL IA] and [30–40 mL PA]	Total	36.08 ± 10.06	19.15 ± 12.04
						Pain	7.15 ± 3.09	3.04 ± 2.76
						Stiffness	3.08 ± 2.24	1.50 ± 1.44
						Function	25.85 ± 7.88	14.62 ± 9.65
					HA (32)[10 mg]	Total	24.81 ± 17.25	15.86 ± 14.78
						Pain	4.90 ± 2.93	3.19 ± 3.04
						Stiffness	2.52 ± 1.83	1.10 ± 1.22
						Function	17.38 ± 15.99	11.57 ± 11.64
				NRS	Prolotherapy (44)[5 mL IA] and [30‐40 mL PA]	4.85 ± 1.71		1.46 ± 1.30
					HA (32)[10 mg]	3.84 ± 1.53		1.86 ± 1.52
Pishgahi, A. et al (2020)^34^	Iran	RCT (92)[4 W]	57.9029.40NR	WOMAC	Prolotherapy (30)[2 mL DX] and [2 mL BW] and [1 ml LD]	65.93 ± 1.67		71.76 ± 2.91
					ACS (32)	56.28 ± 3.13		49.53 ± 3.67
					PRP (30)	60.33 ± 3.70		46.67 ± 4.30
				VAS	Prolotherapy (30)[2 mL DX] and [2 ml BW] and [1 mL LD]	67.00 ± 2.50		63.33 ± 2.47
					ACS (32)	61.25 ± 3.44		46.88 ± 4.45
					PRP (30)	61.10 ± 1.21		56.33 ± 1.02
		RCT (92)[26 W]	57.9029.40NR	WOMAC	Prolotherapy (30)[2 ml DX] and [2 ml BW] and [1 mL LD]	65.93 ± 1.67		72.33 ± 2.57
					ACS (32)	56.28 ± 3.13		34.88 ± 3.35
					PRP (30)	60.33 ± 3.70		45.67 ± 3.82
				VAS	Prolotherapy (30)[2 ml DX] and [2 ml BW] and [1 mL LD]	67.00 ± 2.50		63.30 ± 2.92
					ACS (32)	61.25 ± 3.44		55.00 ± 2.27
					PRP (30)	61.10 ± 1.21		35.00 ± 3.51
Hosseini, B. et al (2019)^35^	Iran	RCT (104)[2 W]	61.2030.70NR	WOMAC	Prolotherapy (52)[10 mL DX]	52.70 ± 9.80		83.70 ± 12.70
					HA (52)[2.5 mL]	55.90 ± 10.4		88.50 ± 15.60
				VAS	Prolotherapy (52)[10 mL DX]	7.80 ± 1.40		2.50 ± 1.10
				HA (52)[2.5 mL]	8.20 ± 1.70		2.10 ± 0.60
Rabago, D. et al (2013)^36^	USA	RCT (90)[52 W]	56.8031.906.6	WOMAC	Prolotherapy (30)[5 ml DX Intra‐articular][7.5 mL DX Extra‐articular][5 mL lidocaine]	Total		15.32 ± 3.32
						Pain		14.18 ± 3.62
						Stiffness		15.55 ± 4.66
						Function		16.25 ± 3.39
					Saline (29)	Total		7.59 ± 3.36
						Pain		7.38 ± 3.67
						Stiffness		9.97 ± 4.72
						Function		5.46 ± 3.44
					Exercise (31)	Total		8.24 ± 3.33
						Pain		9.24 ± 3.63
						Stiffness		8.31 ± 4.68
						Function		7.31 ± 3.40
				KPS	Prolotherapy (30)[5 mL DX Intra‐articular][7.5 mL DX Extra‐articular][5 mL lidocaine]	Frequency score		−1.20 ± 0.21
						Severity score		−0.92 ± 0.21
					Saline (29)	Frequency score		−0.60 ± 0.21
						Severity score		−0.32 ± 0.21
					Exercise (31)	Frequency score		−0.40 ± 0.21
						Severity score		−0.11 ± 0.21
Po‐Jung Pan, et al (2022)^37^	Taiwan	RCT (12)[10 W]	NRNR0.5	WOMAC	Prolotherapy (12)[6 mL DX]	Total	53.2 ± 14.7	27.0 ± 11.6
						Pain	10.3 ± 3.1	4.8 ± 1.9
						Stiffness	4.2 ± 1.3	2.7 ± 1.8
						Function	38.8 ± 10.9	19.5 ± 9.0
Rahimzadeh, P. et al(2018)^38^	Iran	RCT (42)[24 W]	64.328.3NR	WOMAC	Prolotherapy (PRL) (21)[7 mL 25% DX]	Total	67.1 ± 7.9	38.7 ± 6.6
						Pain	14.6 ± 1.4	8 ± 1.6
						Stiffness	5.2 ± 1.3	3 ± 0.7
						Function	47.3 ± 6.7	27.8 ± 5.2
					PRP (21) injection[7 mL PRP solution]	Total	67.9 ± 7.3	31.4 ± 10.2
						Pain	14.8 ± 1.5	6.2 ± 2.1
						Stiffness	5.4 ± 1.2	2.5 ± 0.8
						Function	47.8 ± 4.7	22.8 ± 7.9
Rabago, D. et al (2016)^43^	USA	RCT (22)[52 W]	56.5NR5.6	WOMAC	Prolotherapy	Total	61.0 ± 12.3	80.9 ± 12.6
						Pain	65.4 ± 13.1	82.7 ± 14.9
						Stiffness	57.1 ± 19.2	76.7 ± 16.5
						Function	60.5 ± 12.8	83.4 ± 11.9
Rezasoltani, Z. et al (2020)^44^	Iran	RCT (112)[12 W]	64.832.46.3	KOOS	DX Prolotherapy (28)[8 mL 20% DX Intra‐articular][2 mL 2% lidocaine Intra‐articular]	Pain	11.6 ± 6.8	
						Stiffness	1.0 ± 2.2	
						Function	22.2 ± 16.1	
						Quality of life	5.5 ± 3.0	
					Physical therapy (28)	Pain	9.2 ± 5.3	
						Stiffness	1.9 ± 1.5	
						Function	8 ± 16.3	
						Quality of life	3.8 ± 3.7	
					Botulinum neurotoxin (29)[250 Units Dysport diluted with 5 mL NS]	Pain	11.6 ± 6.7	
						Stiffness	0.5 ± 2.4	
						Function	20.5 ± 18.0	
						Quality of life	3.1 ± 3.4	
					HA (27)[2 mL HA]	Pain	2.1 ± 9.9	
						Stiffness	1.8 ± 2.0	
						Function	2.8 ± 19.6	
						Quality of life	1.7 ± 4.5	
Wing Shan Sit, R. et al (2020)^40^	Hong Kong	RCT (76)[52 W]	62.8 (5.8)24.0 (3.4)9.7	WOMAC	Prolotherapy (38)[5 mL of 25% DX intra‐articular][1 mL of 1% lidocaine HCL]Comparison GroupNormal saline (38)[5 mL]	Pain	–10.34 (–19.20 to –1.49)	
						Stiffness	–8.01 (–18.56 to 2.54	
						Function	–9.55 (–17.72 to –1.39)	
						composite	–9.65 (–17.77 to –1.53)	
Alketa T. Sert et al (2020)^41^	Turkey	RCT (62)[18 W]	55.7 ± 6.630.0 ± 4.625.6 ± 7.9	WOMAC	Prolotherapy (21)[5 mL injection of 25% dextrose solution intra, extra‐articular, 3 times][2.5 mL 1% lidocaine]exercise	Pain	13.7 ± 3.0	6.4 ± 2.6
						Stiffness	5.4 ± 1.1	2.7 ± 1.2
						Function	49.0 ± 7.9	23.5 ± 8.1
						Total	68.7 ± 11.4	32.7 ± 11.6
					Saline (22)intra‐articular [2.5 mL 0.9% sodium chloride +2.5 mL 1% lidocaine] and extra‐articular [5 mL 0.9% sodium chloride +5 mL 1% lidocaine]exercise	Pain	12.9 ± 3.2	9.4 ± 3.4
						Stiffness	5.9 ± 1.7	3.9 ± 1.6
						Function	50.1 ± 13.4	34.0 ± 10.8
						Total	69.2 ± 17.6	46.7 ± 13.5
					Control (19)Only exercise	Pain	14.4 ± 3.4	11.4 ± 2.6
						Stiffness	5.4 ± 1.6	4.2 ± 1.1
						Function	49.0 ± 8.2	44.0 ± 8.5
						Total	68.9 ± 11.9	59.8 ± 10.7
				VAS	Prolotherapy (21)[5 mL injection of 25% dextrose solution intra, extra‐articular, 3 times][2.5 mL 1% lidocaine]Exercise	Pain	7.2 ± 1.0	1.1 ± 1.9
					Saline (22)intra‐articular [2.5 mL 0.9% sodium chloride +2.5 mL 1% lidocaine] and extra‐articular [5 mL 0.9% sodium chloride +5 mL 1% lidocaine]exercise	Pain	7.4 ± 2.0	4.6 ± 1.8
					Control (19)Only exercise	Pain	7.0 ± 0.9	4.5 ± 2.0
Hsieh, R. et al (2021)^42^	Taiwan	RCT (randomized double‐blind Trial) (104)[24 W]	62.4 ± 10.427.2 ± 5.4NR	WOMAC	Prolotherapy (52)[3.5 mL of 50% dextrose intra‐articular][3.5 mL of 2% lidocaine][2‐mL 10 mg/dL HA injection]	Pain	230.8 ± 97.9	80.3 ± 77.9
						Stiffness	100.4 ± 40.6	90.6 ± 40.6
						Function	523.5 ± 318.1	529.8 ± 292.7
					Control (52)[3.5 mL of normal saline][3.5 mL of 2%lidocaine][2‐mL 10 mg/dL HA injection]	Pain	216.9 ± 89.54	199.6 ± 91.9
						Stiffness	105.2 ± 39.6	97.8 ± 42.8
						Function	513.5 ± 326.8	540.9 ± 298.2
				KOOS	Prolotherapy (52)[3.5 mL of 50% dextrose intra‐articular][3.5 mL of 2% lidocaine][2‐mL 10 mg/dL HA injection]	Pain	40.9 ± 16.5	47.4 ± 19.5
						Quality of life	20.7 ± 17.2	24.5 ± 16.0
					Control (52)[3.5 mL of normal saline][3.5 mL of 2%lidocaine][2‐mL 10 mg/dL HA injection]	Pain	42.5 ± 19.5	43.8 ± 20.5
						Quality of life	19.0 ± 18.2	22.5 ± 19.1

BMI: Body Mass Index, OA: Osteoarthritis, RCT: Randomized Control Trial, WOMAC: Western Ontario McMaster Universities Index, IA: Intra‐Articular, PA: Peri‐Articular, DX: Dextrose, BW: Bacteriostatic Water, LD: Lidocaine, ASC: Autologous Conditioned Serum, PRP: Platelet‐rich Plasma, KPS: Knee Pain Scale, HA: Hyaluronic acid, NR: not reported, SF‐36 MCS: Shot Form‐36 Physical Component Summery, SF‐36 MCS: Short form‐36 Mental Component Summery, KOOS: Knee Injury and Osteoarthritis Outcome Score

### 3.1 Mean of WOMAC tool and its subscales

The results of the meta‐analysis, combining studies that used the WOMAC tool, revealed the following findings:

#### 3.1.1 Function

**Figure 2:**
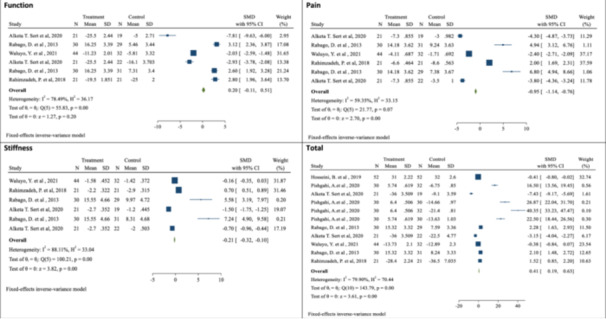
Forest plot of the effect of prolotherapy (3–9 mL with 25%–50% dextrose) on the WOMAC score in patients with knee osteoarthritis.

Patients with KOA who received prolotherapy showed an average increase of 0.20 in function compared with other treatments, although this difference was not statistically significant (SMD: 0.20; 95% CI: −0.11, 0.51; *p* value > 0.05). The heterogeneity among the studies was high (*I* ^2^: 78.49%; *p*_heterogeneity_ < 0.001) (Figure 2).

#### 3.1.2 Pain

The mean pain in patients who received prolotherapy decreased by 0.95 compared with patients who received other treatments or placebo, and this difference was statistically significant (SMD: −0.95; 95% CI: −1.14, −0.76; *p* value < 0.001). The heterogeneity among the studies was moderate (*I* ^2^: 59.35%; *p*_heterogeneity_ = 0.070) (Figure 2).

#### 3.1.3 Stiffness

Patients with KOA who received prolotherapy experienced a statistically significant decrease in stiffness, with a mean reduction of 0.21 (SMD: −0.21; 95% CI: −0.32, −0.10; *p* value < 0.001). The heterogeneity among the studies was high (*I* ^2^: 88.11%; *p*_heterogeneity_ < 0.001) (Figure 2).

#### 3.1.4 Overall WOMAC score

The SMD of the overall WOMAC score increased by 0.41 in patients with KOA who received prolotherapy, indicating improvement. This difference was statistically significant (SMD: 0.41; 95% CI: 0.19, 0.63; *p* value < 0.001). The heterogeneity among the studies was high (*I* ^2^: 79.90%; *p*_heterogeneity_ < 0.001) (Figure 2). Subgroup analysis revealed variations in the impact of prolotherapy based on increasing BMI, age, follow‐up period, and duration of KOA. Specifically, patients with lower BMI demonstrated a more pronounced reduction in mean pain (SMD: −1.26; 95% CI: −1.87, −0.65; *p* value < 0.001), function (SMD: −0.79; 95% CI: −1.36, −0.23; *p* value < 0.001), and joint stiffness (SMD: −0.66; 95% CI: −1.11, −0.21; *p* value < 0.001), as assessed by the WOMAC questionnaire, compared with those with higher BMI. Additionally, the effectiveness of prolotherapy differed concerning age and duration of KOA. Moreover, subgroup analysis based on the duration of follow‐up after intervention demonstrated that follow‐up periods of 20 weeks or less yielded a notable reduction in mean pain (SMD: −3.82; 95% CI: −4.34, −3.29; *p* value < 0.001) and alleviated joint stiffness (SMD: −1.11; 95% CI: −1.46, −0.76; *p* value < 0.001) among individuals with KOA (Table 3).

**Table 3:** The mean difference and related effect size of included randomized control trials.

Outcomes	Scales	Variables	Subgroups	SMD (% 95 CI)	Within groups			Between groups	
					*I* ^2^(%)	Q	*p*_heterogeneity_	*Q*	*p*
WOMAC	Function	Follow up length	≤20 weeks	−2.64 (−3.09, −2.19)	78.88	13.88	<0.00	66.33	<0.00
			>20 weeks	2.82 (2.39, 3.29)	0.00	1.01	0.60		
		Duration of KOA	≤20 years	0.63 (0.26, 1.00)	77.89	15.30	<0.00	45.30	<0.00
			>20 years	−3.81 (−4.58, −3.04)	61.95	11.75	<0.00		
		BMI	≤30 kg/m^2^	−0.79 (−1.36, −0.23)	68.99	21.14	<0.00	16.89	<0.00
			>30 kg/m^2^	0.63 (0.26, 1.00)	77.00	17.30	<0.00		
		Age	≤60 years	0.85 (0.42, 1.27)	65.89	20.88	<0.00	19.69	<0.00
			>60 years	−0.57 (−1.04, −0.11)	87.90	33.00	<0.00		
	Pain	Follow up length	≤20 weeks	−3.82 (−4.34, −3.29)	26.67	2.84	0.25	3.02	0.52
			>20 weeks	1.88 (1.52, 2.26)	28.99	3.02	0.22		
		Duration of KOA	≤20 years	0.33 (−0.02, 0.69)	63.82	26.91	<0.00	13.29	<0.00
			>20 years	−4.26 (−5.03, −3.48)	0.00	0.55	0.46		
		BMI	≤30 kg/m^2^	−1.26 (−1.87, −0.65)	71.00	27.55	<0.00	19.41	<0.00
			>30 kg/m^2^	0.33 (−0.02, 0.68)	63.43	26.99	<0.00		
		Age	≤60 years	0.31 (−0.05, 0.67)	26.98	4.00	0.77	2.17	0.77
			>60 years	−1.04 (−1.62, −0.46)	25.99	3.27	0.40		
	Stiffness	Follow up length	≤20 weeks	−1.11 (−1.46, −0.76)	94.56	36.77	<0.00	17.00	<0.00
			>20 weeks	1.52 (1.17, 1.87)	53.42	4.29	0.12		
		Duration of KOA	≤20 years	0.61 (0.31, 0.91)	92.86	32.38	<0.00	13.08	<0.00
			>20 years	−2.23 (−2.79, −1.67)	91.36	11.58	<0.00		
		BMI	≤30 kg/m^2^	−0.66 (−1.11, −0.21)	97.90	33.44	<0.00	21.22	<0.00
			>30 kg/m2	0.61 (0.31, 0.91)	93.82	32.39	<0.00		
		Age	≤60 years	0.17 (−0.15, 0.49)	97.43	33.56	<0.00	0.25	0.61
			>60 years	0.30 (−0.09, 0.69)	96.94	31.94	<0.00		
	Total	Follow up length	≤20 weeks	−0.58 (−0.86, −0.31)	91.65	36.99	<0.00	11.43	<0.00
			>20 weeks	2.24 (1.87, 2.61)	93.18	21.47	<0.00		
		Duration of KOA	≤20 years	0.92 (0.60, 1.24)	79.88	14.87	0.049	13.02	<0.00
			>20 years	−4.04 (−4.38, −3.25)	58.00	10.33	0.33		
		BMI	≤30 kg/m^2^	0.52 (0.02, 1.02)	29.00	9.03	0.55	0.24	0.62
			>30 kg/m^2^	0.38 (0.13, 0.62)	46.55	12.02	0.20		
		Age	≤60 years	1.41 (1.03, 1.80)	38.00	2.09	0.67	7.89	<0.00
			>60 years	−0.09 (−0.36, 0.18)	31.00	1.88	0.88		
VAS	Total	BMI	≤30 kg/m^2^	1.85 (1.39, 2.32)	83.99	13.22	0.01	12.34	<0.00
			>30 kg/m^2^	3.20 (2.61, 3.78)	–	–	–		
		Follow up length	≤20 weeks	1.89 (1.49, 2.29)	88.72	31.92	<0.00	31.98	<0.00
			>20 weeks	4.68 (3.80, 5.55)	67.80	44.99	<0.00		

Abbreviations: CI, confidence interval, KO, knee osteoarthritis; SMD, standard mean difference.

#### 3.1.5 Publication bias

The results of the Egger's test indicated the presence of publication bias in the combined studies related to function based on the WOMAC tool (*B*: −6.22; SE: 1.39; *p* value < 0.001). However, after conducting a trim and fill analysis to assess the impact of publication bias on the estimated findings, it was determined that bias did not significantly affect the calculated result (SMD: 0.205; 95% CI: −0.11, 0.52). For the mean pain estimation based on the WOMAC tool, Egger's test results indicated no publication bias in the combined pain studies (*B*: 1.53; SE: 0.80; *p* value > 0.05). In the combined studies related to stiffness based on the WOMAC tool, Egger's test revealed the presence of publication bias (*B*: 3.97; SE: 0.762; *p* value < 0.001). However, the trim and fill analysis indicated that the bias had a minimal impact of 0.02 on the calculated result (SMD: −0.23; 95% CI: −0.34, 0.13). Regarding the combination of studies reporting the total WOMAC score, publication bias was detected (*B*: −34.43; SE: 0.511; *p* value < 0.001). The trim and fill analysis showed that publication bias had an effect of 0.02 on the calculated result (SMD: 20.57; 95% CI: 20.40, 20.74). These results suggest that although publication bias was observed in some sections, its impact on the overall findings of the meta‐analysis was minimal for function, pain, stiffness, and the total WOMAC score.

#### 3.1.6 Meta‐regression analysis

The results of the meta‐regression analysis showed with increasing age, the prolotherapy effect on the overall WOMAC score decreased by 0.54 (*B*: −0.54; SE: 1.74; *p* value > 0.05; 95% CI: −3.96, 2.87). Also, with the increase in the BMI, this effect significantly decreased by 12 units (*B*: −12.66; SE: 8.82; *p* value > 0.05; 95% CI: −29.96, 4.63). The results of each WOMAC scale also showed for the function scale, with increasing age, the prolotherapy effect on function increased by 0.40 (*B*: 0.40; SE: 0.53; *p* value > 0.05; 95% CI: −0.64, 1.46). Also, with increasing the BMI, this effect increased (*B*: 3.80; SE: 3.44; *p* value > 0.05; 95% CI: −2.95, 10.56). For the pain scale, with increasing age, the prolotherapy effect on pain increased by 0.39 (*B*: 0.39; SE: 0.43; *p* value > 0.05; 95% CI: −0.46, 1.24). Also, with increasing the BMI, this effect increased (*B*: 1.50; SE: 3.18; *p* value > 0.05; 95% CI: −4.72, 7.74). For the stiffness scale, with increasing age and BMI, the prolotherapy effect on stiffness increased by 0.29 (*B*: 0.29; SE: 0.25; *p* value > 0.05; 95% CI: −0.19, 0.79) and 1.78 (*B*: 1.78; SE: 1.09; *p* value > 0.05; 95% CI: −1.70, 5.28), respectively.

The duration of KOA and the duration of follow‐up after the intervention are two effective variables in determining the prolotherapy effect on patients with KOA. The prolotherapy effect on the function of patients with KOA decreased with increasing the KOA history (*B*: −0.28; SE: 0.15; *p* value > 0.05; 95% CI: −0.59, 0.01). Also, with the increase in the duration of follow‐up after prolotherapy intervention, its effect on the function of patients with KOA increased (*B*: 0.16; SE: 0.08; *p* value < 0.05; 95% CI: 0.00, 0.33).

The prolotherapy effect on pain in patients with KOA decreased with increasing the KOA history (*B*: −0.17; SE: 0.11; *p* value > 0.05; 95% CI: −0.44, 0.06). Also, with the increase in the follow‐up period after the prolotherapy intervention, its effect on the pain of patients with KOA increased (*B*: 0.12; SE: 0.07; *p* value > 0.05; 95% CI: −0.01, 0.27).

Also, the meta‐regression results showed the prolotherapy effect on stiffness with the increase in the KOA history and the duration of the follow‐up period after the treatment decreased (*B*: −0.15; SE: 0.06; *p* value < 0.05; 95% CI: −0.27, −0.02) and increased (*B*: 0.07; SE: 0.04; *p* value > 0.05; 95% CI: −0.02, 0.16), respectively. Finally, the prolotherapy effect on the overall WOMAC score decreased with the increase in the disease history and the duration of the follow‐up period after the intervention (*B*: −0.29; SE: 0.11; *p* value < 0.05; 95% CI: −0.51, −0.08) and (*B*: −0.10; SE: 0.28; *p* value > 0.05; 95% CI: −0.65, 0.45).

### 3.2 Mean pain based on VAS score

**Figure 3:**
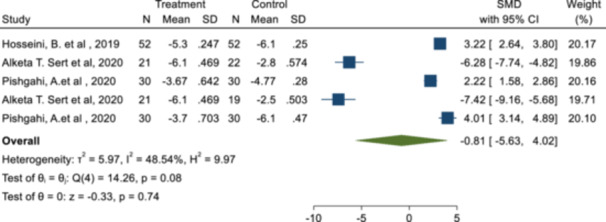
Forest plot of the effect of prolotherapy (3–9 mL with 25 injections to 50% dextrose) on the VAS score in patients with knee osteoarthritis.

In the present meta‐analysis, three studies with seven effect sizes (SMD) reported the mean pain in the two groups of prolotherapy (injection of 3–9 mL of 25%–50% dextrose) and control. The two pain differences based on VAS were reported as percentages or in units of 100 while the rest of the effect sizes reported the mean pain in the range of 0 to 10. Therefore, the two effect sizes reported based on the units of 100 were excluded from the research and the rest of the studies with the reported effect sizes were combined together. The meta‐analysis result showed the mean pain as an SMD decreased 0.81 units out of 10 (SMD: −0.81; 95% CI: −5.63, 4.10; *p*_SMD_ > 0.05; *I* ^2^: 48.54%; *p*_heterogeneity_ = 0.08) (Figure 3, Table 3).

#### 3.2.1 Publication bias

The results of the Eggers test showed the publication bias occurred in the results related to the combination of studies determining the prolotherapy effect on the mean pain (*B*: −19.88; SE: 4.13; *p* value < 0.001). Because the number of articles was small, funnel plot was not used.

#### 3.2.2 Meta‐regression analysis

The results of meta‐regression analysis showed with increasing age, the prolotherapy effect on the mean pain based on VAS significantly increased by 1.95 units out of 10 (*B*: 1.94; SE: 0.85; *p* value < 0.05; 95% CI: 0.26, 3.61) while this effect decreased with increasing the BMI (*B*: −1.62; SE: 5.82; *p* value > 0.05; 95% CI: −13.04, 9.78).

The prolotherapy effect on the mean pain in patients with KOA was evaluated based on the disease history and the duration of the follow‐up period after the intervention using meta‐regression. The number of studies reporting the mean history of KOA in patients was small. Therefore, meta‐regression analysis was not performed for this variable, but for the follow‐up duration, the results showed the prolotherapy effect on the mean pain in patients with KOA increased with the increase in the follow‐up period after the intervention (B: 0.16; SE: 0.54; *p* value > 0.05; 95% CI: −0.89, 1.22).

### 3.3 Mean of KOOS tool and its subscales

**Figure 4:**
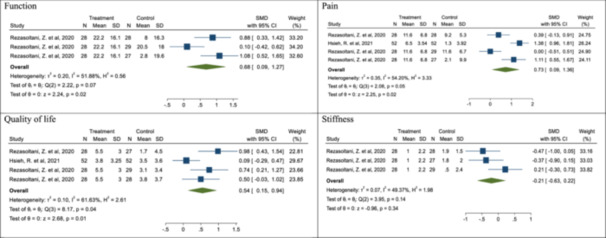
Forest plot of the effect of prolotherapy (3–9 mL with 25%–50% dextrose) on the KOOS score in patients with knee osteoarthritis.

The results showed after combining the studies which used the KOOS tool, function in patients with KOA, who received prolotherapy significantly increased by 0.68 compared with other treatments (in one study with three different effect sizes) (SMD: 0.68; % 95 CI: 0.09, 1.27; *p*_SMD_ < 0.05; *I* ^2^: 51.88%; *p*_heterogeneity_ > 0.05) (Figure 4). The mean pain based on this questionnaire significantly increased by 0.73 after combining four effect sizes from two studies (SMD: 0.73; 95% CI: 0.09, 1.38; *p*_SMD_ < 0.05; *I* ^2^: 54.20%; *p*_heterogeneity_ > 0.05) (Figure 4).

The mean quality of life in patients with KOA, receiving prolotherapy after combining four effect sizes in two studies increased by an average of 0.54 compared with other treatments (SMD: 0.54; 95% CI: 0.15, 0.94; *p*_SMD_ < 0.05; *I* ^2^: 61.63%; *p*_heterogeneity_ < 0.05) (Figure 4). The mean stiffness in the patients who received prolotherapy decreased by an average of 0.21 (SMD: −0.21; 95% CI: −0.63, 0.22; *p*_SMD_ > 0.05; *I* ^2^: 49.37%; *p*_heterogeneity_ > 0.05) (Figure 4).

Because the number of studies in the combination of the results of the mean KOOS tool and its scales was small, often only one study in the scale analyzes, meta‐regression analysis and publication bias evaluation were not performed.

### 3.4 Quality assessment results

**Figure 5:**
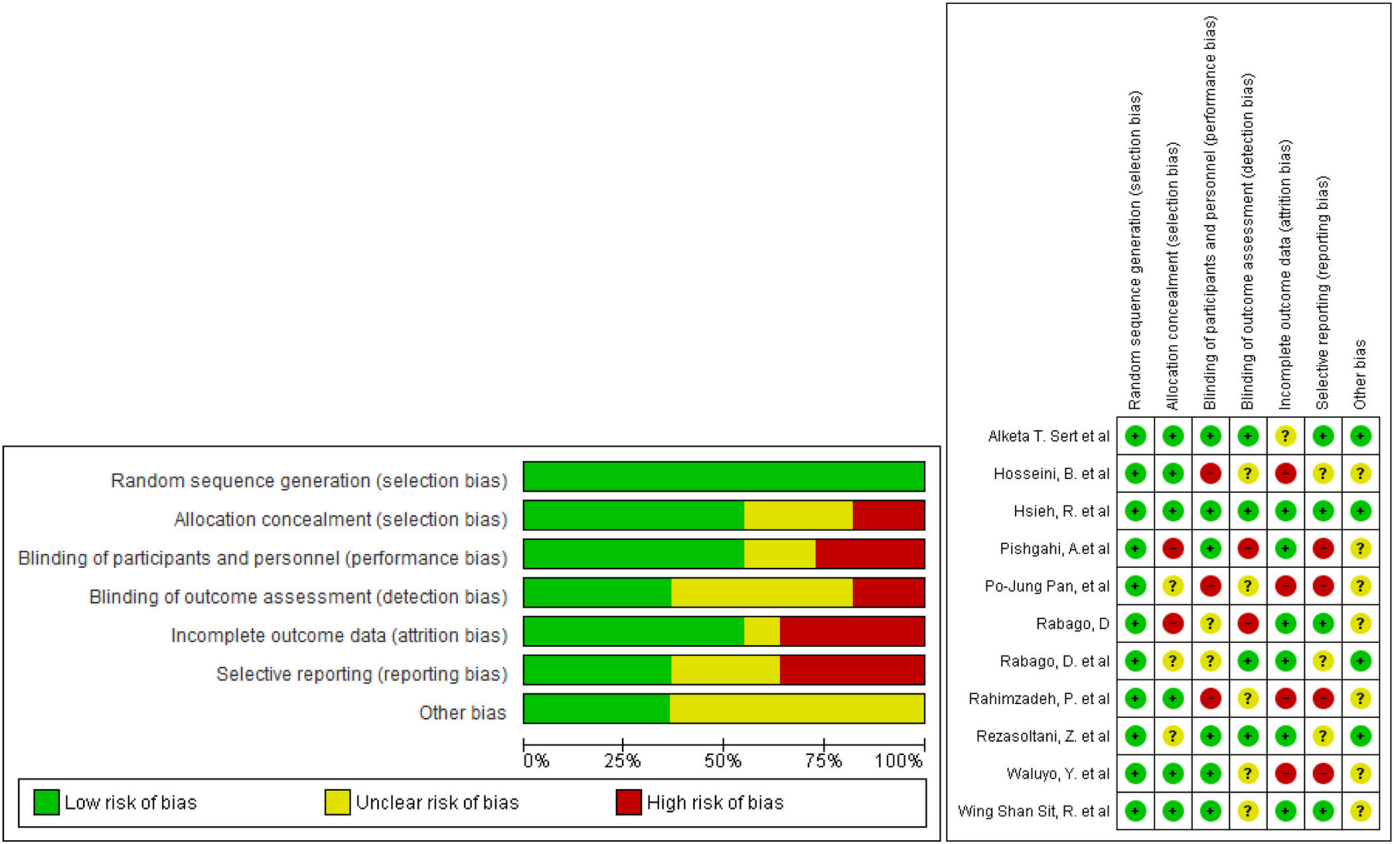
Risk of bias graph: review authors' judgments about each risk of bias item presented as percentages across and judgments about each risk of bias item for each all‐included study.

The results of the quality assessment, conducted using the Cochrane checklist, indicated that the majority of the selected RCTs were of high quality. The results indicated that two studies were categorized as having selection and detection bias, three studies were categorized as having performance bias, and four studies were categorized as having attrition bias and selective bias. Please refer to Figure 5 for a visual representation of the quality assessment results.

## 4 DISCUSSION

The study conducted a comprehensive systematic review and meta‐analysis on the impact of prolotherapy in patients with KOA. While the precise mechanisms of prolotherapy on soft tissues and joints are not fully understood, previous research has proposed several potential mechanisms.^22^, ^24^, ^36^, ^45^, ^46^ One mechanism is the reduction of local inflammation, where the injection of a proliferative solution may aid in decreasing inflammation and promoting healing, potentially alleviating symptoms like pain and stiffness in KOA patients. Another suggested mechanism is the promotion of proliferation in joints or soft tissues, with prolotherapy solutions like dextrose stimulating tissue growth and repair, leading to improved joint function. Additionally, prolotherapy has been linked to pain reduction by modulating pain signals and providing analgesic effects through injections into the affected area. Prolotherapy works in the treatment of KOA by inducing an inflammatory response through the injection of a solution, typically containing substances like dextrose and lidocaine, near the painful or damaged joints. Dextrose, a type of sugar, can indeed induce an inflammatory response when administered, particularly in the short term. When used in prolotherapy, dextrose solutions can stimulate an inflammatory reaction that triggers the body's natural healing processes. This inflammatory response is essential for the repair and regeneration of tissues like tendons, ligaments, and cartilage. Studies have shown that dextrose concentrations above 10% can operate through inflammatory mechanisms, while lower concentrations are considered noninflammatory. The inflammatory response induced by dextrose in prolotherapy is a crucial part of the treatment process, as it promotes the production of growth factors necessary for tissue repair and growth. Therefore, while dextrose may create inflammation in the short term, it plays a beneficial role in stimulating tissue healing and regeneration in the context of prolotherapy. This process initiates the production of fibroblasts, which are the body's repair cells, leading to the deposition of new tissue fibers that repair the injury, stabilize the area, restore function, and reduce pain. The inflammatory cascade triggered by prolotherapy is believed to promote tissue proliferation and remodeling, aiding in the healing process.^25^, ^36^, ^47^, ^48^ This can lead to decreased pain levels and increased comfort for individuals with KOA. Ultimately, the improvements in pain, function, and overall quality of life observed in patients who receive prolotherapy contribute to increased satisfaction with the treatment. While the precise mechanisms underlying these effects require further investigation, prolotherapy has shown promise as a therapeutic option for individuals with KOA.^20^, ^27^, ^49^, ^50^, ^51^ Indeed, one of the hypotheses regarding the effect of prolotherapy on joints and tissues is that it promotes soft tissue proliferation, leading to tissue repair, recovery, and a reduction in edema and inflammation. Studies have demonstrated that dextrose 20% has a regenerative effect on healing damaged Achilles tendons by promoting fibroblast proliferation. Additionally, in a high glucose environment, there is an increase in the production of platelet‐derived growth factors by vascular endothelial and mesangial cells. While these functions have been observed in laboratory experiments, the practical application of these findings in human samples still presents challenges. The translation of laboratory findings to clinical settings involves complexities such as the variability of patient response, the influence of other factors in the healing process, and the need for further validation in controlled clinical trials. It is important for future research to continue investigating the mechanisms of action underlying prolotherapy and to address these challenges in human samples. This will help establish a clearer understanding of how prolotherapy affects joints and tissues, and potentially improve its effectiveness as a therapeutic approach for conditions like KOA.^52^ The second hypothesis regarding the effect of dextrose in prolotherapy is that it has a prechondrogenic effect. This hypothesis suggests that dextrose may promote the formation of new cartilage. However, the available evidence on this topic is conflicting, and there is no consensus among researchers.^53^ For example, a systematic review highlighted the positive and significant beneficial effects of dextrose prolotherapy in the treatment of symptomatic KOA, suggesting its potential in the management of chronic musculoskeletal conditions. In addition, research has shown that dextrose solutions can stimulate the production of growth factors essential for the repair and growth of tendons, ligaments, and other soft tissues, indirectly supporting the idea of a prechondrogenic effect. It is important to note, however, that while the regenerative potential of dextrose in prolotherapy has been demonstrated, the claim of direct cartilage regeneration must be approached with caution.^50^, ^54^, ^55^ The third hypothesis regarding the effect of prolotherapy is related to its modulating effect on pain. However, there is conflicting information regarding this hypothesis as well. The results of the present meta‐analysis can contribute to reducing these contradictions and shedding light on the effect of prolotherapy on pain in KOA patients. According to the results of the meta‐analysis, after combining selected clinical trial studies, there was an increase in function among KOA patients who received prolotherapy, as assessed by the WOMAC questionnaire. Prolotherapy involves the injection of a proliferative solution into damaged or inflamed joints, which aims to stimulate and accelerate the body's natural healing process. The increased speed of the repair process can potentially lead to an improvement in knee joint function. While pain modulation is a key aspect of prolotherapy, the specific mechanisms by which prolotherapy exerts its pain‐relieving effects are not yet fully understood. It is believed that prolotherapy injections may have analgesic effects by reducing inflammation and promoting tissue healing, which can ultimately lead to a decrease in pain experienced by patients with KOA. The findings of the meta‐analysis support the notion that prolotherapy can positively impact knee joint function in patients with OA. However, it is important to note that individual responses to prolotherapy may vary, and further research is needed to better understand the underlying mechanisms and optimize treatment protocols for pain modulation in KOA patients.^40^, ^53^ Research suggests that dextrose prolotherapy may be as effective as, or possibly more effective than, HA, PRP, and ACS in improving functional outcomes in patients with KOA. Specifically, studies have shown that dextrose prolotherapy was more effective in improving functional outcomes in patients with generalized KOA compared with alternative treatments such as saline, exercise, local corticosteroid (LC) injections, and pulsed radiofrequency (PRF) therapy. Additionally, in terms of pain relief, dextrose prolotherapy has been found to be more effective in reducing pain compared with other treatments including saline, exercise, LC injections, PRF therapy, HA injections, and physical therapy (PT). While some studies have shown promising results for HA, PRP, ACS, and exercise programs in improving functional outcomes, dextrose prolotherapy has demonstrated similar or superior efficacy in reducing pain and improving functional outcomes for KOA patients.^22^, ^24^, ^56^

The meta‐regression results showed the prolotherapy effect on knee function increases with increasing age and BMI because they are two important risk factors in the occurrence of KOA. Also, the results of meta‐regression in the present meta‐analysis showed the prolotherapy effect on knee function increased over time after prolotherapy injection in patients. Therefore, the more time passes after prolotherapy injection in patients with KOA, their knee function improves which can be due to the increase in the strength of the ligaments. The results of the present meta‐analysis showed the mean pain based on the WOMAC and VAS tools in these patients significantly decreased compared with the ones who received other treatments or placebo. In patients with KOA, pain is caused due to the destruction of the cartilages of the bone heads, the reduction of the joint space, the damage of the ligaments, and finally the development of edema and inflammation. Prolotherapy reduces pain in patients by reducing edema and inflammation and repairing soft tissues and ligaments. This effect on pain reduction in patients is greater with increasing age, and the BMI, as well as the passage of time after prolotherapy injection. Stiffness significantly reduces in patients treated by prolotherapy injection. With increasing BMI and age, this effect decreases, but the important point is that the more time passes after prolotherapy, its effect on stiffness increases. Finally, in patients with KOA, the bones are reaching each other due to the destruction of the joint cartilage, and their erosion occurs in the joint space which leads to pain, swelling, stiffness, and reduced performance. Prolotherapy injection can act as a stimulus for the production of new tissues and ultimately repair and improve the existing tissues, which over time has a greater effect on reducing pain and other consequences in patients.^38^

In 2020, a meta‐analysis was conducted by Tze Chao Wee et al.^25^ to determine the effect of dextrose prolotherapy on KOA, which included very few clinical trial studies, and its reported charts had low power. The present study included more studies in the meta‐analysis and reported the prolotherapy effect with better power. On the other hand, in this study, various tools such as WOMAC, VAS, and KOOS were examined and evaluated. Therefore, the current meta‐analysis reported more accurate and up‐to‐date information than studies published in the past. Another advantage of this study is that it investigates the duration of prolotherapy follow‐up and the intervention's long‐term effect in KOA patients. According to this meta‐analysis, prolotherapy can reduce pain and stiffness for up to 20 weeks. However, beyond this point, the intervention's efficacy in patients tends to decline.

The study refrained from conducting subgroup analyses based on characteristics such as age, BMI, length of follow‐up after prolotherapy, prolotherapy dosage, or disease duration. This decision might have been influenced by the limited number of research studies available within each subgroup, rendering it challenging to ascertain precise connections or correlations. Additionally, a notable limitation of the study lies in the absence of a specific comparison group to assess the effects of prolotherapy. Instead of juxtaposing prolotherapy against a single treatment, the meta‐analysis extended its evaluation to encompass a diverse array of other treatments, including PRP, HA, and placebo.

## 5 CONCLUSION

This thorough analysis indicates that dextrose prolotherapy could provide advantages in decreasing joint pain and rigidity while enhancing knee performance in individuals dealing with OA. Nevertheless, it is crucial to understand that various factors such as the patient's age, BMI, medical history, duration of follow‐up post prolotherapy, and the dosage given can impact the efficacy of this treatment. It is wise to thoroughly evaluate these factors before giving prolotherapy injections. Moreover, to enhance our comprehension of the efficacy of prolotherapy in comparison to alternative treatments like PRP and HA injections, it is suggested that a multicenter clinical trial with a larger number of participants be carried out. Furthermore, future research should consider including postintervention follow‐up periods to help make better decisions about how long the effects of prolotherapy last. These research endeavors could offer crucial understandings and assist in developing evidence‐driven recommendations for prolotherapy treatment of KOA.

## AUTHOR CONTRIBUTIONS

**Sorour Khateri**: Conceptualization; validation; visualization; writing—review & editing; data curation; supervision; resources; project administration. **Fatemeh Behbahani Nejad**: Writing—review & editing; visualization; writing—original draft; software; formal analysis; resources; supervision; data curation. **Farnoush Kazemi**: Data curation; supervision; resources; formal analysis; software; visualization; writing—review & editing; writing—original draft. **Behnaz Alaei**: Investigation; writing—original draft; writing—review & editing; validation. **Mobin Azami**: Writing—original draft; writing—review & editing; visualization; software; formal analysis; data curation; supervision; resources. **Asra Moradkhani**: Data curation; supervision; resources; formal analysis; software; visualization; writing—review & editing; writing—original draft. **Lobat Majidi**: Investigation; conceptualization; writing—original draft; writing—review & editing; visualization; validation; software; formal analysis; project administration; data curation; supervision; resources. **Yousef Moradi**: Methodology; validation; visualization; writing—review & editing; project administration; formal analysis; software; resources; supervision; data curation; writing—original draft; investigation; conceptualization.

## CONFLICT OF INTEREST STATEMENT

The authors declare no conflict of interest.

## TRANSPARENCY STATEMENT

The lead author Yousef Moradi affirms that this manuscript is an honest, accurate, and transparent account of the study being reported; that no important aspects of the study have been omitted; and that any discrepancies from the study as planned (and, if relevant, registered) have been explained.

## Data Availability

Data and materials are available within the complementary materials, and further information can be available by request to the corresponding author.
